# Differences in cardiac phenotype and natural history of laminopathies with and without neuromuscular onset

**DOI:** 10.1186/s13023-019-1245-8

**Published:** 2019-11-19

**Authors:** Raffaello Ditaranto, Giuseppe Boriani, Mauro Biffi, Massimiliano Lorenzini, Maddalena Graziosi, Matteo Ziacchi, Ferdinando Pasquale, Giovanni Vitale, Alessandra Berardini, Rita Rinaldi, Giovanna Lattanzi, Luciano Potena, Sofia Martin Suarez, Maria Letizia Bacchi Reggiani, Claudio Rapezzi, Elena Biagini

**Affiliations:** 1 Cardiology Unit, Cardio-Thoracic-Vascular Department, Sant’Orsola-Malpighi Hospital, University of Bologna, Via G. Massarenti 9, 40138 Bologna, Italy; 2 Cardiology Division, Department of Biomedical, Metabolic and Neural Sciences, University of Modena and Reggio Emilia, Policlinico di Modena, Modena, Italy; 3 University College London Institute for Cardiovascular Science and Barts Heart Centre, St. Bartholomew’s Hospital, London, UK; 4 Neurology Unit, Sant’Orsola-Malpighi University Hospital, Bologna, Italy; 5 Italian National Research Council (CNR), Institute of Molecular Genetics IGM Bologna, Bologna, Italy

**Keywords:** Lamin, Emerin, Neuromuscular disorders, Atrial fibrillation, Bradyarrhythmias, Ventricular tachycardias, Familial cardiomyopathies

## Abstract

**Objective:**

To investigate differences in cardiac manifestations of patients affected by laminopathy, according to the presence or absence of neuromuscular involvement at presentation.

**Methods:**

We prospectively analyzed 40 consecutive patients with a diagnosis of laminopathy followed at a single centre between 1998 and 2017. Additionally, reports of clinical evaluations and tests prior to referral at our centre were retrospectively evaluated.

**Results:**

Clinical onset was cardiac in 26 cases and neuromuscular in 14. Patients with neuromuscular presentation experienced first symptoms earlier in life (11 vs 39 years; *p* <  0.0001) and developed atrial fibrillation/flutter (AF) and required pacemaker implantation at a younger age (28 vs 41 years [*p* = 0.013] and 30 vs 44 years [*p* = 0.086] respectively), despite a similar overall prevalence of AF (57% vs 65%; *p* = 0.735) and atrio-ventricular (A-V) block (50% vs 65%; *p* = 0.500). Those with a neuromuscular presentation developed a cardiomyopathy less frequently (43% vs 73%; *p* = 0.089) and had a lower rate of sustained ventricular tachyarrhythmias (7% vs 23%; *p* = 0.387). In patients with neuromuscular onset rhythm disturbances occurred usually before evidence of cardiomyopathy. Despite these differences, the need for heart transplantation and median age at intervention were similar in the two groups (29% vs 23% [*p* = 0.717] and 43 vs 46 years [*p* = 0.593] respectively).

**Conclusions:**

In patients with laminopathy, the type of disease onset was a marker for a different natural history. Specifically, patients with neuromuscular presentation had an earlier cardiac involvement, characterized by a linear and progressive evolution from rhythm disorders (AF and/or A-V block) to cardiomyopathy.

## Introduction

Laminopathies are a group of inherited conditions due to mutations in the *LMNA* gene, that encodes the nuclear envelope proteins lamin A and C, via alternate splicing [1]. Laminopathies are characterized by a high phenotypic heterogeneity including heart disease, neuromuscular disorders, premature aging and metabolic disorders [2–5]. *LMNA* associated cardiac and skeletal muscle disease - often coexisting in the same patient - are the most frequent clinical manifestations. The spectrum of cardiac involvement ranges from supraventricular tachyarrhythmias and/or conduction system disease to dilated cardiomyopathy (DCM) and ventricular tachyarrhythmias [6–12]. Sudden cardiac death may occur due to bradyarrhythmias or to malignant ventricular arrhythmias [13], even in the presence of mild left ventricle systolic dysfunction. Similarly, *LMNA*-related neuromuscular disorders are characterized by a wide heterogeneity in clinical manifestations, Emery Dreifuss muscular dystrophy (EDMD) being the most common phenotype. EDMD is typically characterized by early onset joint contractures with slowly progressive scapulo-humero-peroneal muscle weakness, and can be caused by mutations in genes other than *LMNA*, mainly *EMD* that encodes emerin.

Although clinical manifestation in patients with *LMNA* mutations have been extensively described, the exact time course of cardiological and neuromuscular disease and their relation remain unclear. Specifically, it is not known if a neuromuscular onset is associated with a different cardiac phenotype or cardiac disease progression. The aim of this study was therefore to investigate differences in cardiac phenotype and natural history in relation to the presence of neuromuscular involvement at presentation, in patients with a diagnosis of laminopathy. Furthermore, in order to test the hypothesis that neuromuscular presentation (phenotype) per se might be associated with a specific cardiac natural history, irrespective of genetics, we compared patients with neuromuscular presentation and LMNA or EMD mutations.

## Methods

In this observational study, we prospectively evaluated all *LMNA* mutation carriers from a single Italian centre (S.Orsola-Malpighi University Hospital, Bologna), between December 1998 and November 2017. We also retrospectively examined clinical, ECG and echocardiographic reports available prior to evaluation at our centre. According to the presence of signs and/or symptoms of skeletal myopathy at clinical presentation (not necessarily in our centre), patients were divided into two groups: “with neuromuscular onset” and “without neuromuscular onset”. Data of 7 patients with *EMD*-related disease were also recorded. All patients underwent periodical clinical, electrocardiographic and echocardiographic monitoring.

DCM was defined as the presence of left ventricular (LV) dilation and systolic dysfunction in the absence of abnormal loading conditions (hypertension or valve disease) or coronary artery disease sufficient to cause global systolic impairment [14]. “Hypokinetic non-dilated cardiomyopathy” (HNDC) was defined as LV ejection fraction (EF) < 45% or biventricular global systolic dysfunction in absence of dilatation [15]. Restrictive cardiomyopathy was defined as a non-dilated LV with normal wall thickness and EF, and severe diastolic dysfunction with restrictive filling pattern, elevated filling pressures and dilated atria [14]. Sustained ventricular tachyarrhythmias (SVT) were defined as ventricular tachyarrhythmias with a rate ≥ 120/min, lasting > 30 s.

A neurological involvement was investigated on the basis of personal history and/or symptoms including joint contractures, muscle weakness or wasting, orthopaedic surgery, and exercise tolerance. Electromyography (EMG), muscle imaging or muscle biopsy were performed in selected cases. Patients underwent periodical neurological evaluation, even when no skeletal muscle involvement was recorded at first evaluation. Elevation of serum creatine kinase (CK) in isolation was not considered diagnostic of neuromuscular involvement in absence of clinical, EMG, imaging or histological evidence of skeletal myopathy.

A detailed family history was collected for each proband in order to identify other potentially affected family members. A genetic diagnosis was made by DNA sequencing from peripheral blood and mutations were considered pathogenic if previously described in literature, in the presence of co-segregation or based on the site and the type of mutation. After mutation identification, cascade genetic screening was performed in family members, following informed consent.

Continuous data distribution was assessed with the Shapiro-Wilk test and expressed as median and interquartile range (IQR). Data were compared by the Fisher’s exact test for proportions, and Mann Whitney test for continuous variables. Clinical events were reported as counts and percentages (i.e. events/total number of patients × 100). Data were collected following informed consent.

## Results

Of the 41 *LMNA* mutation carriers, 40 were clinically affected. Table 1 reports clinical, ECG and echocardiographic characteristics at first evaluation at our centre. Fourteen patients were assigned to the group “with neuromuscular onset” and 26 to the group “without neuromuscular onset”. A single *LMNA* mutation carrier, who did not have any cardiac or neuromuscular phenotypic expression at baseline or during follow up, was excluded from the analysis.

**Table 1:** Characteristics at first clinical evaluation at our centre of patients with *LMNA* mutations with and without neuromuscular onset

	Overall	With neurological Onset	Without neurological onset	*P* value
Number of patients	40	14 (35)	26 (65)	
Number of families	31	12 (39)	19 (61)	
Males	22 (55)	7 (50)	15 (58)	0.744
Age at symptom onset, yrs	33 (21–42)	11 (8–30)	39 (32–46)	< 0.0001
Age at first contact at our center, yrs	39 (29–74)	31 (20–44)	43 (36–49)	
Follow-up duration, months	30 (6–70)	24 (12–101)	32 (8–63)	
Diagnostic pathway				
Signs or symptoms		12 (86)	19 (73)	0.452
Screening		2 (14)	7 (27)	
Cardiomyopathy	24 (60)	5 (36)	19 (73)	0.040
Dilated CMP	13 (32)	2 (14)	11 (42)	0.089
Hypokinetic non dilated CMP	8 (20)	2 (14)	6 (23)	0.688
Restrictive CMP	2 (5)	0	2 (8)	0.533
Right ventricle CMP	1 (2)	1 (7)	0	0.350
History of atrial fibrillation	17 (42)	6 (43)	11 (42)	1.000
Sinus node dysfunction	4 (10)	3 (21)	1 (4)	0.114
Atrio-ventricular block	23 (57)	6 (43)	17 (65)	0.197
1st degree	8 (20)	2 (14)	6 (23)	
2nd degree	7 (17)	2 (14)	5 (19)	
3rd degree/high degree	8 (20)	2 (14)	6 (23)	
Positive familial history for				
Sudden death	7 (17)	3 (21)	4 (15)	0.678
PM implantation (high degree AVB)	11 (27)	3 (21)	8 (30)	0.715
CMP	16 (40)	5 (36)	11 (42)	0.746
NYHA class III- IV	7 (17)	4 (28)	3 (12)	0.214
PM recipients	8 (20)	2 (14)	6 (23)	0.688
ICD recipients	8 (20)	1 (7)	7 (27)	0.221

Values are expressed as N, N (%) or median (interquartile range)

*LMNA, EMD* gene codifying for lamin A/C and emerin, respectively, *ICD* implantable cardioverter defibrillator, *PM* pacemaker, *AVB* atrio-ventricular block, *NYHA* New York Heart Association

### Patients with neuromuscular onset

Among the 14 patients in this group males and females were equally represented. *LMNA* mutations were: 12 missense, 1 splice site, and 1 deletion (Table 2). Median age at first evaluation at our Centre was 31 years (IQR 20–44). The diagnosis of laminopathy occurred following familial screening in 2 patients. At time of genetic diagnosis, overt neuromuscular involvement was present in all cases whereas none had cardiac involvement. Eleven patients (79%) were diagnosed with EDMD in the first or second decade of life and 3 (21%) were diagnosed with a non-specific myopathy in the third decade; median age at diagnosis was 11 years (IQR 8–30).

**Table 2:** Genetics of *LMNA* mutated patients with neurological onset (*N* = 14)

	Gene	Location	Nucleotide Change	Protein Change	Predicted Effect
**Family 1**					
F; 16 yo	LMNA	Exon 4	c.746G > A	p.Arg249Gln	Missense
**Family 2**					
F; 50 yo	LMNA	Exon 9	c.1580G > C	p.Arg.527.Pro	Missense
**Family 3**					
M; 38 yo	LMNA	Exon 11	c.1930C > T	p.Arg644Cys	Missense
M; 38 yo	LMNA	Exon 11	c.1930C > T	p.Arg644Cys	Missense
**Family 4**					
F; 46 yo	LMNA	Exon 3 Exon 4	c.569G > A; c. 746G > A	p.Arg190Gln p.Arg249Gln	Missense Missense
**Family 5**					
F; 34 yo	LMNA	Exon 1	c.203_208 (delAGGTGG)	p.Glu68_Val69 del	Deletion
**Family 6**					
M; 52 yo	LMNA	Exon 4	c.746G > A	p.Arg249Gln	Missense
**Family 7**					
M; 46 yo	LMNA	Exon 9	c.1567G > A	p.Gly523Arg	Missense
**Family 8**					
M; 17 yo	LMNA	Exon 4	c.775 T > G	p.Tyr259Asp	Missense
**Family 9**					
M; 19 yo	LMNA	Exon 4	c.746 G > A	p.Arg249Gln	Missense
**Family 10**					
F; 29 yo	LMNA	Intron 9	c.1608 + 1G > T	–	Splice site
**Family 11**					
F; 22 yo	LMNA	Exon 1	c.188 T > A	p.Ile63Asn	Missense
F; 19 yo	LMNA	Exon 1	c.188 T > A	p.Ile63Asn	Missense
**Family 12**					
M; 27 yo	LMNA	Exon 4	c.746G > A	p.Arg249Gln	Missense

*M* male, *F* female, *yo* years old. The age reported refers to first contact at our centre

**Fig. 1:**
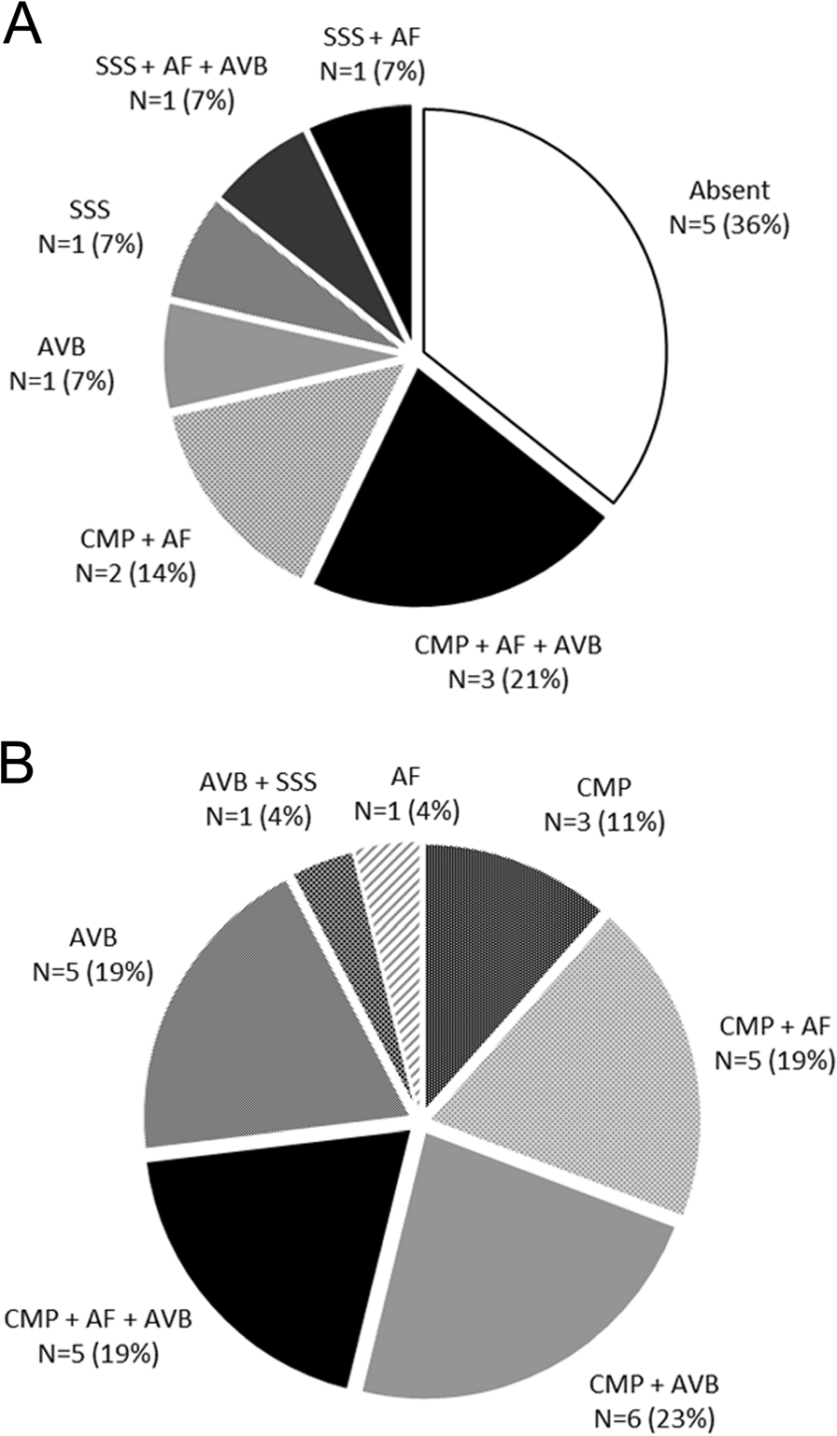
**a** Cardiac phenotype spectrum at first clinical evaluation at our centre of patients with *LMNA* mutations and neurological onset (*N* = 14). SSS: sick sinus syndrome; AVB: atrio-ventricular block; CMP: cardiomyopathy; AF atrial fibrillation/flutter. **b** Cardiac phenotype spectrum at first clinical evaluation at our centre of patients with *LMNA* mutations without neurological onset (*N* = 26). SSS: sick sinus syndrome; AVB: atrio-ventricular block; CMP: cardiomyopathy; AF atrial fibrillation/flutter

**Fig. 2:**
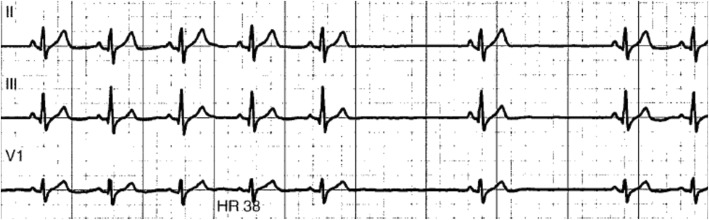
Second degree sino-atrial block with 2:1 conduction ratio in a 22-year old female, with Emery Dreifuss muscular dystrophy due to p.Ile63Asn missense *LMNA* mutation

Nine patients (64%) developed cardiac involvement prior to referral to our centre (Fig. 1a summarizes the spectrum of cardiac phenotypic expression). All had rhythm disturbances: 2 (14%) had atrial fibrillation (AF), 1 (7%) atrio-ventricular (A-V) block, 1 (7%) sinus node dysfunction, and 5 (36%) had a combination of brady- and atrial tachyarrhythmias (Fig. 2). Cardiomyopathy was also present in 5 patients (36%): 2 DCM, 2 HNDC and 1 isolated right ventricular cardiomyopathy mimicking arrhythmogenic right ventricular cardiomyopathy. Patients with LV cardiomyopathy had a severe LV dysfunction (LVEF ≤35%) and 3 had biventricular involvement.

Two patients (14%) had previously undergone pacemaker (PM) implantation for A-V block, while a single patient had a primary prevention implantable cardiac resynchronization therapy defibrillator (CRT-D). Serum CK levels were raised in 65% of the patients, with a mean abnormal value of 631 UI/L.

### Patients without neuromuscular onset

Fifteen (58%) of the 26 patients were males, median age at first evaluation at our centre was 43 years (IQR 36–49). Diagnosis was made due to rhythm disturbances or heart failure symptoms in most cases (*n* = 19, 73%). Seven patients (27%) were identified after family screening. *LMNA* mutations were: 16 missense, 7 splice site, 2 deletion and 1 frameshift (Table 3).

**Table 3:** Genetics and cardiac manifestations of *LMNA* mutated patients without neurological onset (*N* = 26)

	Gene	Location	Nucleotide Change	Protein Change	Predicted Effect
**Family 1**					
M; 53 yo	LMNA	Exon 6	c.1004G > A	p.Arg335Gln	Missense
M; 26 yo	LMNA	Exon 6	c.1004G > A	p.Arg335Gln	Missense
**Family 2**					
F; 47 yo	LMNA	Exon 7	1370delA	p.Lys457SerfsX2	Deletion
**Family 3**					
M; 19 yo	LMNA	Exon 6	c.1003G > A	p.Arg335Glu	Missense
**Family 4**					
F; 55 yo	LMNA	Exon 11	c.1912G > A	p.Gly638Arg	Missense
**Family 5**					
M; 54 yo	LMNA	Exon 7	c.1202G > A	p.Arg401His	Missense
**Family 6**					
M; 42 yo	LMNA	Exon 1	n/a	p.Arg72Alafs*24	Frame shift
**Family 7**					
F; 51 yo	LMNA	Exon 4	c.752A > C	p.Gln251Pro	Missense
**Family 8**					
M; 46 yo	LMNA	Exon 9	c.1517 A > C	p.His506Pro	Missense
**Family 9**					
F; 38 yo	LMNA	Intron 9	c1608 + 1G > T	–	Splice site
M; 35 yo	LMNA	Intron 9	c1608 + 1G > T	–	Splice site
**Family 10**					
F; 29 yo	LMNA	Exon 3	c.548 T > C	p.Leu183Pro	Missense
**Family 11**					
F; 41 yo	LMNA	Exon 6	c.1007G > A	p.Arg336Gln	Missense
**Family 12**					
M; 60 yo	LMNA	Intron 1	c.357-1G > A IVS1-1G > A	–	Splice site
F; 46 yo	LMNA	Intron 1	c.357-1G > A IVS1-1G > A	–	Splice site
F; 49 yo	LMNA	Intron 1	c.357-1G > A IVS1-1G > A	–	Splice site
F; 49 yo	LMNA	Intron 1	c.357-1G > A IVS1-1G > A	–	Splice site
M; 21 yo	LMNA	Intron 1	c.357-1G > A IVS1-1G > A	–	Splice site
**Family 13**					
M; 38 yo	LMNA	Exon 6	c.1129C > T	p.Arg377Cys	Missense
**Family 14**					
M; 50 yo	LMNA	Exon 2	c.481G > A	p.Glu161Lys	Missense
**Family 15**					
M; 46 yo	LMNA	Exon 2	c.466C > A	p.Arg156Ser	Missense
M; 34 yo	LMNA	Exon 2	c.466C > A	p.Arg156Ser	Missense
**Family 16**					
M; 44 yo	LMNA	Exon 4	c.671 C > T	p.Thr224Ile	Missense
**Family 17**					
F; 39 yo	LMNA	Exon 2	c.481G > A	p.Glu161Lys	Missense
**Family 18**					
F; 37 yo	LMNA	Exon 2	c.513G > A	pLys171Asn	Missense
**Family 19**					
F; 36 yo	LMNA	Exon 5	c.855delG	p.Ala287LeufsX191	Deletion

*M* male, *F* female, *yo* years old. The age reported refers to first contact at our centre. *n/a* not available

**Fig. 3:**
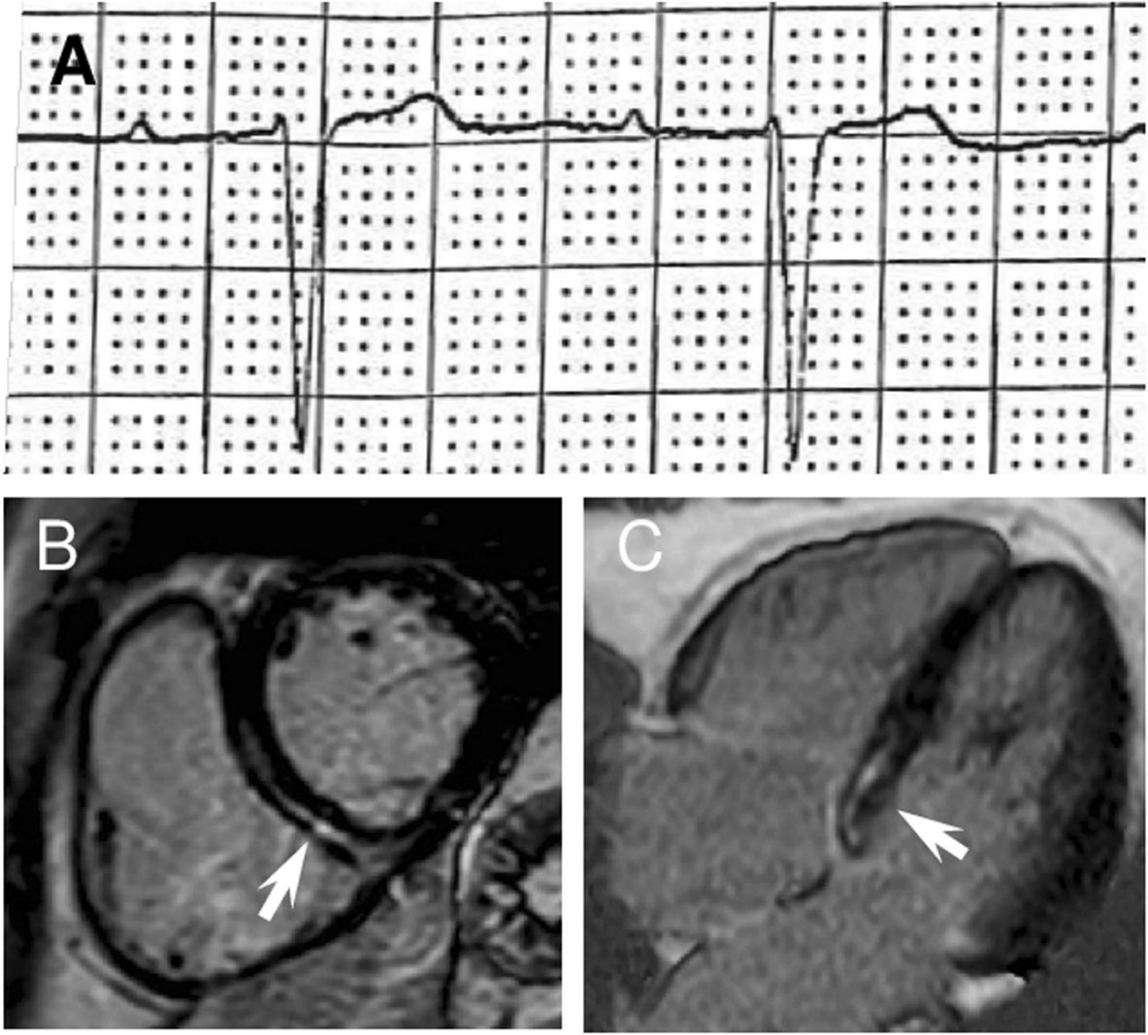
43-year old male with a *LMNA* frameshift mutation without neuromuscular presentation. **a** V1 lead ECG showing first degree atrio-ventricular block, **b-c** cardiac magnetic resonance showing midwall late gadolinium enhancement in the basal interventricular septum. Suggestive for myocardial fibrosis

At first clinical evaluation at our centre, 19 patients (73%) had a cardiomyopathy, in isolation (*n* = 3, 11%) or associated with arrhythmias (AF *n* = 5, 19%; A-V block *n* = 6, 23%; both *n* = 5, 19%; Fig. 3). Seven patients (27%) had arrhythmias in absence of cardiomyopathy: 1 (4%) AF, 5 (19%) A-V block and 1 (4%) a combination of sinus node and A-V node dysfunction. Overall, AF was present in 11 patients (42%). Figure 1b summarizes the spectrum of cardiac phenotypic expression. Six patients (23%) had previously undergone PM implantation for A-V block and 7 patients (27%) had received a primary prevention implantable cardioverter defibrillator (ICD).

**Fig. 4:**
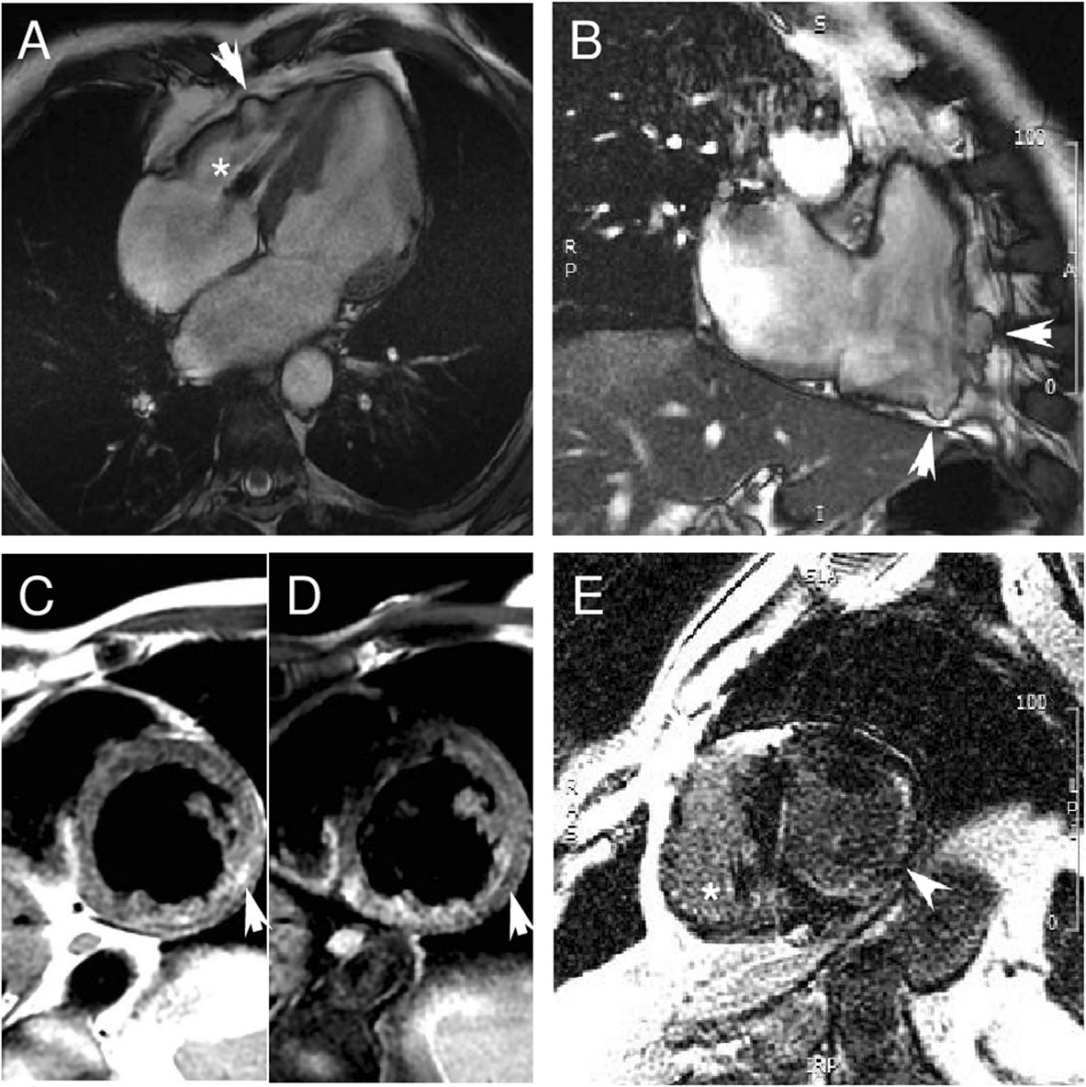
CMR of a 54-year male, carrier of p.Arg401His missense *LMNA* mutation, affected by DCM. (**a-b**) Four chamber and RV long axis SSFP images show biventricular dilation, bulging of the RV free wall (white arrow, panel **a-b**) and diaphragmatic wall (white arrow, panel **b**). (**c**) Two chamber T1-weighted and (**d**) fat suppressed T1-weighted slices showing LV fatty replacement of mid lateral wall (white arrow). (**e**) IR LGE slice showing fibrosis in the infero-lateral wall (with focal transmural pattern) and in the interventricular septum (image quality was due to respiratory artifacts and to the presence of pacemaker [*]). CMR: cardiac magnetic resonance. DCM: dilated cardiomyopathy. SSFP: steady-state free precession. LV-RV: left-right ventricle. IR LGE: inversion recovery late gadolinium enhancement

Cardiomyopathy phenotype included: 11 DCM, 6 HNDC and 2 restrictive cardiomyopathy. Among the 17 patients with LV systolic dysfunction, 7 (41%) had a severe impairment (LVEF ≤35%), 6 (35%) had biventricular involvement and 3 (17%) had increased LV trabeculations. One 54-year old patient with a history of complete A-V block and AF had a biventricular cardiomyopathy with multiple aneurysms in the diaphragmatic and free wall of the right ventricle. Coronary arteries were unobstructed at angiography. Cardiac magnetic resonance showed a severely dilated left ventricle (indexed end diastolic volume: 146 mL/m2) with systolic dysfunction (EF 40%) and mildly dilated right ventricle (indexed end diastolic volume: 111 ml/m2) with reduced EF (40%) and confirmed the wall motion abnormalities (Fig. 4 a-b). Tissue characterization (Fig. 4 c-d) revealed multiple areas of fibro-fatty replacement. In this case, possible phenocopies including desmosomal-related cardiomyopathy and sarcoidosis were excluded by genetic analysis, positron emission tomography, lung CT and, endomyocardial biopsy.

No patient had evidence of neuromuscular involvement. Serum CK levels were raised in 47% of the patients with a mean abnormal value of 217 UI/L.

### Follow-up of patients with neuromuscular onset

Median follow up was 24 months (IQR 12–101). New onset AF was recorded in 2 patients, therefore 57% experienced atrial tachyarrhythmias by the end of the follow-up. In one patient, the atrial conduction disease progressed to atrial paralysis. Three patients underwent PM implantation for A-V block (*n* = 1), sinus node disease (*n* = 1) or both (*n* = 1). A primary prevention CRT-D was implanted in a patient with new onset HNDC due to positive family history for sudden death, high inducibility of VT on electrophysiological study and moderate LV dysfunction. The patient affected by right ventricular cardiomyopathy received a primary prevention ICD, due to severe right ventricular dysfunction, non-sustained ventricular tachycardias and the need of a pacing for sinus and A-V node dysfunction. Thereafter he experienced an appropriate ICD activation and a progression towards severe biventricular involvement. No sudden death occurred. Five patients with cardiomyopathy had hospital admissions due to heart failure during follow-up and 4 of them subsequently underwent heart transplantation (median age 43 [IQR 34–48]).

### Follow-up of patients without neuromuscular onset

New onset AF was reported in 6 patients (23%) during a median follow up of 32 months (IQR 8–63); 65% of patients had atrial tachyarrhythmias at the end of follow up. With the exception of 2 patients with atrial flutter – who were treated successfully with cavo-tricuspid isthmus ablation – the attempts of rhythm control with electrical or pharmacological cardioversion were ineffective. No patients underwent pulmonary vein isolation. Atrial paralysis was documented in a single patient. One patient underwent PM implantation due to A-V block. A primary prevention ICD was implanted in 7 patients (4 new implants and 3 device upgrades) and 1 ICD was implanted for secondary prevention. Four of the ICD recipients (50%) received a CRT-D device. During follow up 6 patients (23%) experienced appropriate shocks and/or antitachycardia pacing for ventricular arrhythmias, with an arrhythmic storm in 3 cases. Six (23%) patients underwent cardiac transplantation (median age 46 [IQR 34–53]) due to end stage heart failure (5/6) or to recurrent ventricular arrhythmias (1/6). One patient developed a mild neuromuscular involvement, with muscle atrophy involving the shoulder girdle. Table 4 compares clinical events reported during follow-up in the two groups.

**Table 4:** Clinical events reported during follow-up in *LMNA* mutated patients

	Patients with neurological onset (*N* = 14)	Patients without neurological onset (*N* = 26)	*P* value
New onset of atrial fibrillation (any form)	2 (14)	6 (23)	0.688
PM implantation	3 (21)	1 (4)	0.114
ICD implantation	2 (14)	8 (31)	0.445
SVT/arrhythmic storm	1 (7)	6 (23)	0.387
Admission for heart failure	5 (36)	11 (42)	0.746
Heart transplantation	4 (29)	6 (23)	0.717
Thromboembolic events	0	0	

Values are expressed as N or N (%). *SVT* sustained ventricular tachyarrhythmia, *PM* pacemaker, *ICD* implantable cardioverter defibrillator

### Differences in clinical manifestations between patients with and without neuromuscular onset

**Fig. 5:**
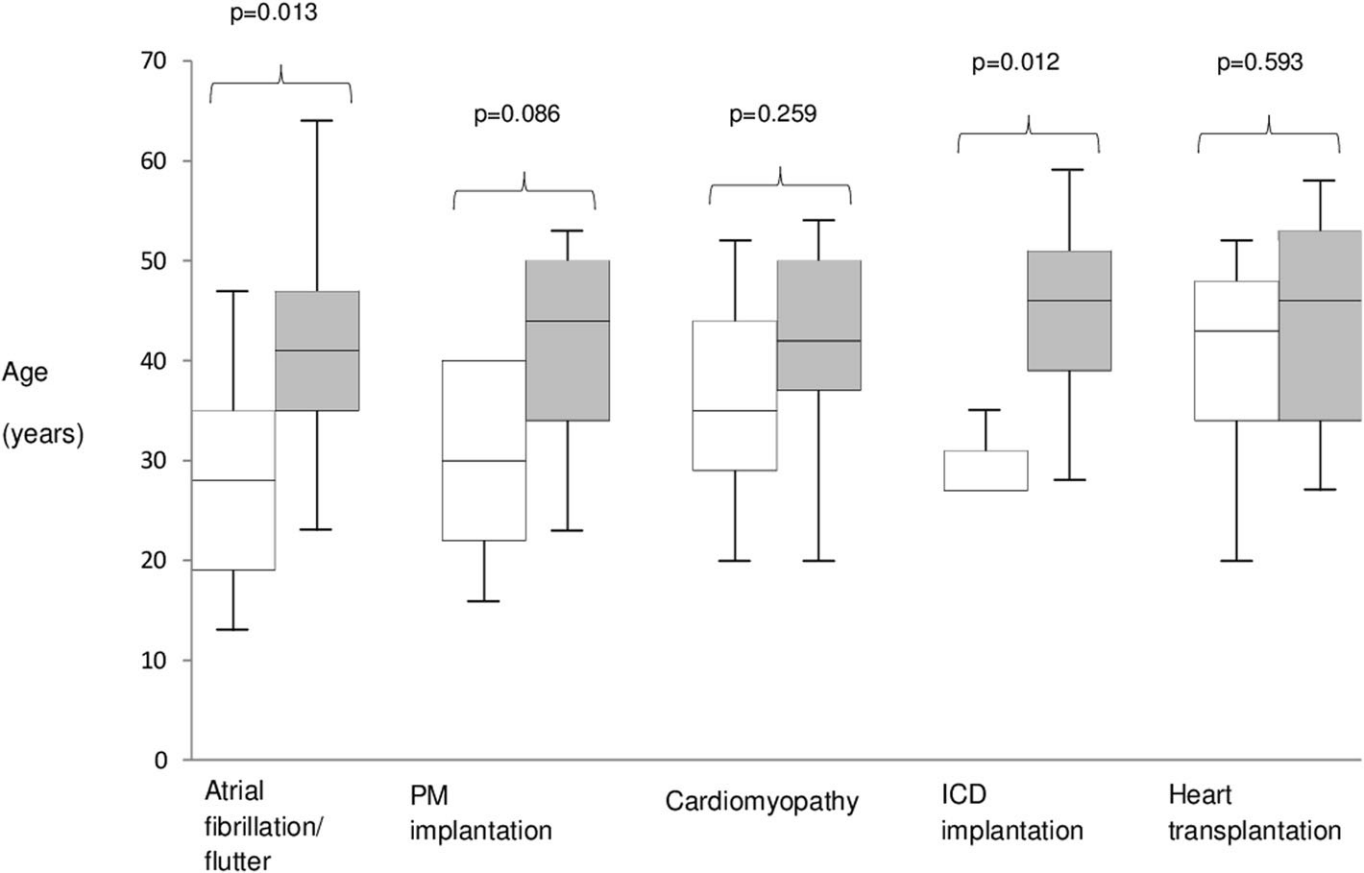
Box and whiskers plot showing age distribution of different clinical events in *LMNA* patients with (white) and without (gray) neuromuscular onset. Middle horizontal line inside box indicates median. Bottom and top of the box are 25th and 75th percentiles, the whiskers indicate the lowest and highest value. PM: pacemaker. ICD: implantable cardioverter defibrillator

Patients with neuromuscular onset had an earlier presentation, during infancy or adolescence in most of the cases (median age 11 years), mainly as EDMD, followed by the first evidence of cardiac disease by a median age of 13 years (IQR 10–15) (maximum timelag 38 years). In patients without neuromuscular onset, first cardiac symptoms occurred later in life, at a median age of 39 years (*p* <  0.0001). Regarding arrhythmias, at the end of the follow-up A-V block (of any degree) and AF had a similar prevalence between the two groups (50% vs 65%, *p* = 0.500 and 57% vs 65%; *p* = 0.735 respectively). Sinus node dysfunction was more frequent in patients with skeletal myopathy (21% vs 4%; *p* = 0.114), whereas atrial paralysis was reported in one patient for each group. Patients with neuromuscular presentation (Fig. 5) experienced earlier AF (age 28 vs 41, *p* = 0.013) and PM implantation (age 30 vs 44; *p* = 0.086). The percentage of patients requiring permanent pacing (including PM recipients and those who received an ICD due to a concomitant indication for prevention of ventricular arrhythmias) was equal in the two groups (42% vs 42%; *p* = 1.000).

Patients without neuromuscular presentation had a higher prevalence of cardiomyopathy (73% vs 43%, *p* = 0.089) and were older at diagnosis (age 42 vs 35, *p* = 0.259). DCM was the dominant phenotype in this group (58% of all cardiomyopathies), whereas DCM and HNDC where equally represented in the other group. The higher prevalence of heart muscle involvement in patients without neuromuscular onset was associated with a higher number of implanted ICDs (58% vs 21%, *p* = 0.045) and a higher burden of SVT (23% vs 7%, *p* = 0.387). Despite this, no significant differences were reported in the prevalence of heart transplantation (23% vs 29%; *p* = 0.717) or in the median recipient age (43 vs 46; *p* = 0.593).

**Fig. 6:**
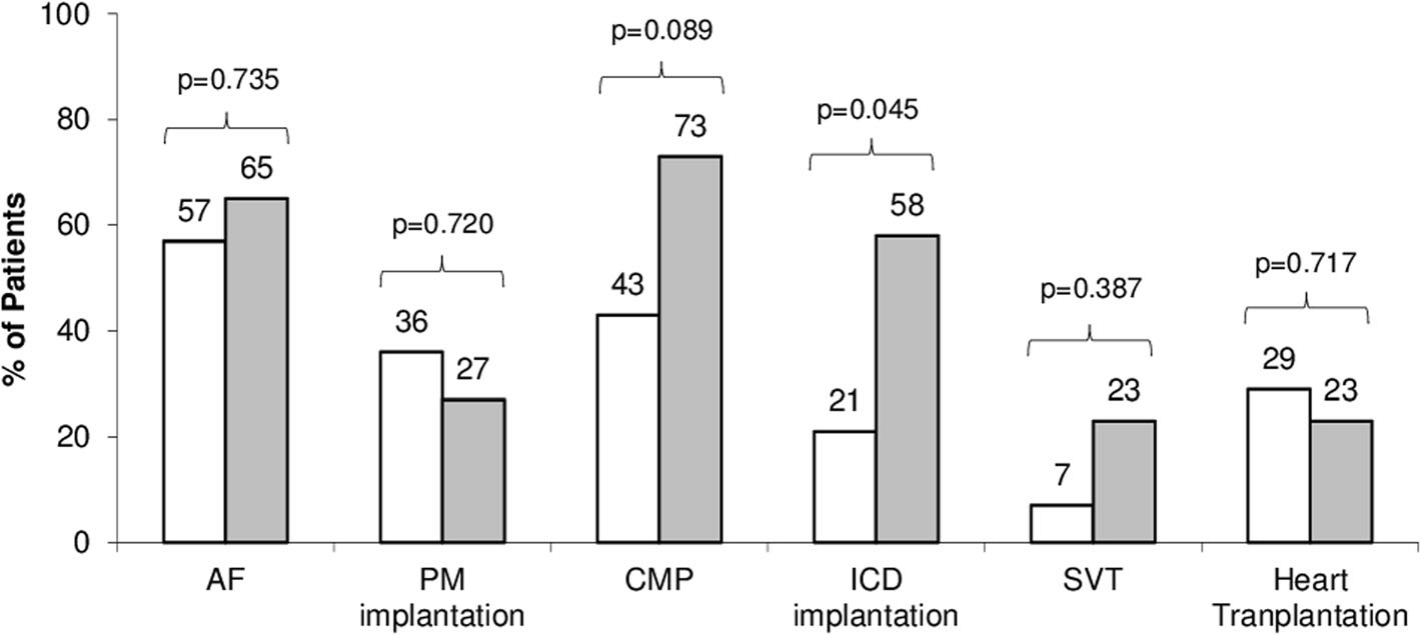
Different overall prevalence at the end of follow-up of clinical events in *LMNA* patients with (white) and without (gray) neuromuscular presentation. AF: atrial fibrillation; PM: pacemaker; CMP: cardiomyopathy; SVT: sustained ventricular tachycardias

All patients with neuromuscular presentation who received a diagnosis of cardiomyopathy had a previous history of rhythm disturbance with the exception of 2 cases, where the diagnosis was concomitant. On the contrary, no pattern of progression from rhythm disturbance to cardiomyopathy was present in those without a neuromuscular presentations: AF and A-V block could precede the diagnosis of cardiomyopathy, be diagnosed at the same time or later. Figure 6 shows the different overall prevalence of clinical events between the two populations.

### Clinical characteristics and follow up of patients with emerinopathy

Seven male patients (4 families) affected by X-linked EDMD were referred to our Centre at a median age of 26 years (IQR 14–30) and followed for a median time of 108 months (IQR 72–172). All had neuromuscular symptoms as first evaluation, with a median age of 6 years (IQR 5–8). At last follow up 6 patients (85%) had cardiac involvement. All developed AF at a median age of 27 [IQR 23–37]) and 5 required PM implantation at a median age of 23 [IQR 22–24] due to A-V block (*n* = 1), sinus node dysfunction (*n* = 1) or both (*n* = 3). A single patient developed cardiomyopathy with mild systolic dysfunction; none had ventricular arrhythmias. The time interval from neurological to cardiac disease onset was of 14,5 years (IQR 14–15). Compared to patients with *LMNA* mutations and a neurological onset, patients with emerinopathy presented a higher burden of AF (85% vs 57%; *p* = 0.337) that occurred an earlier age (27 vs 31 *p* = 0.746), and a higher rate of PM implantation (71 vs 36%; *p* = 0.182) at an earlier age (23 vs 28; *p* = 0.461). Differently, heart muscle involvement was rare (a single case of cardiomyopathy) and no SVT was documented.

## Discussion

The main findings of our study are: 1) neuromuscular onset is a marker for a specific natural history in laminopathy patients. Specifically, these patients have a linear and predictable progression over time, from muscular dystrophy to rhythm disturbances and finally cardiomyopathy. 2) With the exception of sinus node dysfunction, that was more frequent in EDMD patients, the prevalence of A-V block and AF was similar between the two groups, but patients with a neuromuscular presentation had earlier arrhythmias. 3) Prevalence of cardiomyopathy (particularly DCM) and SVT was higher among patients without neuromuscular onset, although the two groups had a similar rate and age of cardiac transplantation.

Most of the patients with neuromuscular presentation (64%) developed cardiac involvement later in life: the delay between neuromuscular and cardiac symptom onset was variable and sometimes very long. These findings suggest that serial reassessment of cardiac status in patients with a diagnosis of EDMD is mandatory. On the other hand, patients without neuromuscular onset did not develop an overt skeletal myopathy (with a single exception). The possibility that these patients could develop neuromuscular involvement in the future cannot however be entirely ruled out due to the limited observation time. Our results are in line with the phenotypic clustering reported by *Benedetti* et al. [16] in a cohort of patients with *LMNA* mutations, where those with childhood onset had an almost exclusively skeletal muscle involvement (predominantly EDMD), while patients with adult onset developed cardiac disorders or muscle weakness with a limb-girdle distribution. With the limitation of the small size of the screened families in our study population and the limited numbers of relatives who carried the mutation, all the affected relatives of probands with neuromuscular presentation had a skeletal muscle involvement as clinical onset. At the same time, all the affected relatives of patients with an exclusive cardiac phenotype had an isolated cardiological involvement. Differently from our findings, *Bonne* et al. and *Brodsky* et al. [17, 18] have previously described the possibility of the coexistence of both phenotypes as clinical onset within the same family.

Our data confirm the high frequency of AF in laminopathies, as well as advanced A-V block, requiring PM implantation at a young age. Although the prevalence of high degree A-V block and AF was similar, irrespective of clinical presentation, patients with neuromuscular onset experienced arrhythmia earlier in life (on average AF and PM implantation occurred more than 10 years earlier). On the other hand, sinus node dysfunction was more frequent in patients with EDMD (21% vs 4%). Regarding heart muscle involvement, patients without neuromuscular onset had a prevalence of cardiomyopathy that was almost twice that of the other group (42% vs 73%; *p* = 0.089), mostly DCM. On the contrary DCM and HNDC were equally distributed in patients with neuromuscular presentation. A progression from HNDC to DCM was not observed in this study, suggesting that they could be the expression of two different pathophysiologic models; however, a limited follow-up duration (median 41 months) and heart failure therapy could have masked this progression. We described two cases, 1 in each group, with a cardiac phenotype that mimicked arrhythmogenic cardiomyopathy. *LMNA* carriers have been described with clinical, morphological and histological phenotypes overlapping with arrhythmogenic cardiomyopathy [19], in the absence of desmosomal gene mutations, with conduction disease being the sole ‘red flag’ for the correct diagnosis. These findings may justify the need to exclude *LMNA* mutations in patients with suspected arrhythmogenic cardiomyopathy, particularly when conduction disease is present.

This study confirms the malignant nature of laminopathies in terms of ventricular arrhythmias and progression to advanced heart failure. In our series sustained SVTs during the follow-up were more frequent in patients without neuromuscular involvement (23% vs 7%) with just 1 EDMD patient experiencing SVT. The low incidence of events in patients with skeletal myopathy differs from previous reports. *Van Rijsingen* et al. [10] reported that in patients with *LMNA* mutations, the diagnosis of muscular dystrophy or a positive family history of muscular dystrophy was not associated with a different incidence of ventricular arrhythmias. The rate of SVT reported by the Authors was of 17% in EDMD patients and 19% in non-EDMD patients. More than 20% of the whole population of this study required cardiac transplantation during follow-up and this is consistent with previous reports. *Hasselberg* et al. [11] reported that 19% of genotyped *LMNA* patients underwent heart transplantation during a follow up of 8 years (median age 46 years). The need for heart transplantation in our series was independent from the involvement of the skeletal muscle and occurred at a median age of 45 years. Differently from what observed for other clinical events, neuromuscular involvement did not lead to an anticipation in the timeline and heart transplant was performed at a similar age in the two groups (median 43 vs 46 years).

**Fig. 7:**
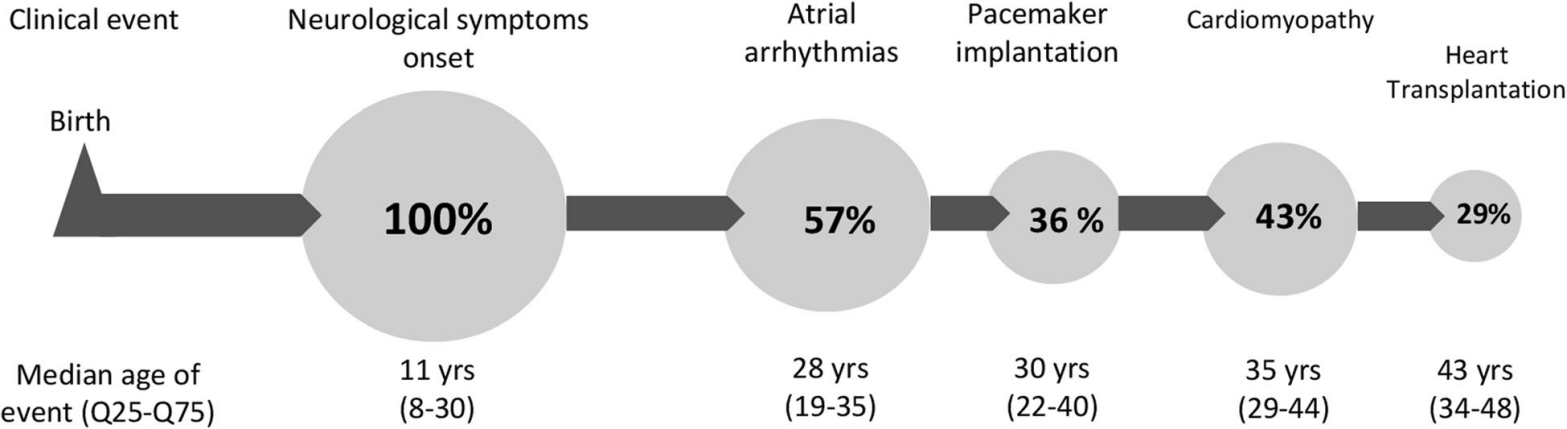
Timeline of clinical events in the lifetime of patients with *LMNA* mutations and neuromuscular onset

In this series, patients with a neuromuscular presentation had a linear predictable progression over time (Fig. 7). Specifically, skeletal myopathy developed first, followed by arrhythmias (A-V block, sick sinus syndrome and AF in various combinations) and eventually, cardiomyopathy. This pattern of progression was not observed in the other patients.

The cardiac involvement in X-linked EDMD patients of our study, compared to patients with LMNA mutations and a neurological onset, was characterized by higher burden of supraventricular tachyarrhyrhmias and bradyarrhythmias that occurred at a younger age and a lower frequency of cardiomyopathy.

## Limitations

Referral bias cannot be excluded since this is a study from a single tertiary centre with a cardiac transplant program and expertise for management of complex ventricular tachycardias.

## Conclusion

In patients with laminopathy, the type of disease onset was a marker for a different natural history. Specifically, patients with neuromuscular presentation had an earlier cardiac involvement, characterized by a linear and progressive evolution from rhythm disorders (AF and/or A-V block) to cardiomyopathy. Prevalence of AF and A-V block was similar, regardless of clinical onset, whereas sinus node dysfunction was more frequent in EDMD patients. Patients with neuromuscular onset had a lower prevalence of cardiomyopathy and ventricular arrhythmias, but a similar prevalence of heart transplantation at a similar age.

## Data Availability

The datasets used and analysed during the current study are available from the corresponding Author on reasonable request.

## Abbreviations

AF: Atrial fibrillation/flutter
AVB: Atrio-ventricular block
CK: Creatine kinase
CRT-D: Implantable cardiac resynchronization therapy defibrillator
DCM: Dilated cardiomyopathy
EDMD: Emery Dreifuss muscular dystrophy
EMG: Electromyography
HNDC: Hypokinetic non dilated cardiomyopathy
HT: Heart transplantation
ICD: Implantable cardioverter defibrillator
IQR: Interquartile range
LV: Left ventricle
LVEF: Left ventricle ejection fraction
NYHA: New York Heart Association
PM: Pacemaker
SVT: Sustained ventricular tachyarrhythmias
